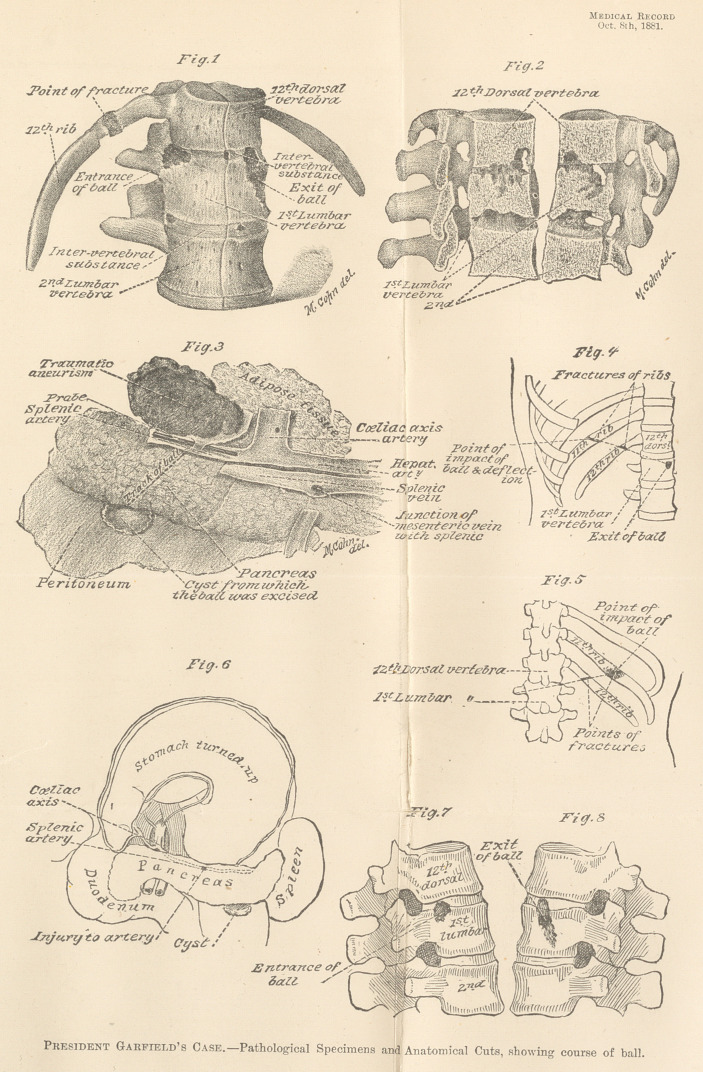# Selections

**Published:** 1881-10

**Authors:** 


### SELECTIONS.

### REPORT OF THE CASE OF PRESIDENT GARFIELD,

### Accompanied With a Detailed Account of the Autopsy.

By D. W. Bliss, M. D., Washington, D. C.,

SURGEON IN CHARGE OF THE CASE.

(*With Illustrations.*)

The great interest which has been manifested by the medical public in the surgical history of the case of President Garfield, and my close and direct connection with it as surgeon in charge, from the time I was summoned until his death, imposes upon me the obligation of giving, even at this early date, a general summary of the salient points connected with its diagnosis, treatment, and pathology.

It seems important at this time, in view of an implied demand on the part of my professional brethren throughout the country, that, at the risk of anticipating the complete and technical report, which will appear in due time under the editorial direction of J. J. Woodward, Surgeon U. S. A., and signed by all the gentlemen associated with me, that I should present such data as may serve to give the leading facts of the general plan of management of the case, the reasons for making the diagnosis, and such other points as were developed in its study which may serve to explain the most important autopsical lesions, the report of which accompanies this paper.

Perhaps these conditions can be fulfilled in no better way than by a summary in which the main and important data are given in the form of a general medical history. It is, perhaps, unnecessary to state at this point that I shall not undertake to reproduce the daily bulletins, nor a minute history of the dietetics, as they are not necessary to enable the profession to comprehend the general treatment as applied to the case in view of the erroneous diagnosis made, or to the conditions presented by the autopsy.

Immediately after the shooting of President Garfield, on the morning of July 2d, I was summoned by the Secretary of War to take charge of the case. I was conducted to an upper room in the building, where I found the President lying upon a mattress, in a semi-prone position, on the left side. He presented the appearance of perfect collapse, the lines of expression were lost, there was extreme pallor, sighing respiration (about eight or ten per minute) ; pulse exceedingly small, feeble, and frequent, and ranging about 120. The ingesta lying upon the mattress indicated that he had recently vomited, and upon mentioning the fact the President replied that he had not ; but assurances from the physicians and others, with the evidence before me, indicated that the emesis had taken place while he was unconscious. Large beads of perspiration stood upon his face, forehead, hands, and forearms.

There were present at that time Dr. Smith Townsend, the Health Officer of the District, and Dr. Purvis. The former, who was the first physician to reach the wounded President, informed me that he had administered half an ounce of brandy and a drachm of aromatic spirits of ammonia internally.

The President's coat had previously been removed ; the remainder of his clothing was intact, except that over the region of the wound, which was so arranged as to expose the point of entrance of the ball.

The President complained of a sense of weight and numbness, and subsequently of a tingling sensation and pain in the lower extremities. With a view of exploring the wound to ascertain the course of the ball and the organs involved in its passage, I introduced a Nélaton probe, which took a direction downward and forward, on a line which would represent a point of exit four inches to the right, and nearly directly opposite to the umbilicus. The point of entrance of the ball, which was oval and sharply cut, was on the right side, four inches from the median line of the spine, and on a line with the eleventh rib. A slight discharge of blood was oozing from this orifice, and had soiled the clothing. I passed the probe in the direction previously indicated, through the tenth intercostal space, for a distance of three and one-half inches from the surface of the body, to what appeared to be a cavity, and I was unable to detect any foreign substance beyond the rib to indicate the presence of fragments of bone or the missile. In attempting to withdraw the probe it became engaged between the fractured fragments and the end of the rib, and could not be liberated until pressure was made upon the sternal end of the rib so as to, slightly elevate its fractured extremity. I then passed the little finger of my left hand to its full extent into the wound, which developed the character and extent of the fracture of the rib, and was only able to reach a point on a line with the inner surface of the rib, where it came in contact with what appeared to be lacerated tissue or comparatively firm coagula, probably the latter. After withdrawing my finger I made an exploration with a long, flexible silver probe, which I suitably curved before entering, and gently passed it downward and forward, and downward and backward in several directions, with a view of indicating the course of the ball, if it had been deflected by contact with the rib, and meeting with resistance from soft parts I desisted, and excluded the probability of deflection, being inclined to the opinion that the ball had entered the liver, which, if true, would not warrant further exploration in that direction.

By this time a large number of physicians had gathered in the room, and I gave to them a hurried account of my examination, and expressed the opinion that no further explorations should be made during the stage of collapse, and that stimulants by the stomach should not at that time be given, as the President was suffering from constant nausea, and in his condition of collapse absorption would not take place, and further that they would become a source of additional irritation. In these opinions, expressed at the council in one corner of the room, the physicians concurred. The gentlemen in attendance at this time, so far as I can recollect, were Drs. Townshend, Purvis, Reyburn, Norris, Lincoln and Ford.

The President repeatedly requested that he be taken to the White House, and after further consultation and a full understanding of the manner and detail of his transfer, his speedy removal was agreed upon. Temporary dressings were applied to the wound, when the President was lifted on to the mattress, carefully placed upon a stretcher, conveyed down-stairs, and placed in an ambulance in waiting. The vehicle was driven with great care over the rough pavement of Sixth street, about forty yards distance, until reaching the smooth asphalt pavement of Pennsylvania avenue. The great rush of people, in the excitement, made it necessary to move rapidly. On the way there was no disagreeable motion in the carriage, which fact is attested by Dr. Townshend and others who accompanied me in the ambulance. On inquiry, the President replied that the motions of the carriage did not give him any discomfort. At the street railroad crossings at Seventh and Fourteenth streets, the vehicle was driven with exceeding caution, and with scarcely an uncomfortable motion. He was then taken in the same manner as before to his room, and placed with extreme caution on the low family bed. The room is known as the southwest or family room of the house. On his arrival thither a careful examination was made of his condition. The pulse continued feeble, frequent, and extremely compressible; the respiration was slow and sighing; extremities and surface cold, with occasional vomiting and profuse perspiration over the entire body; voice husky, with constant complaint of severe pains in the inferior extremities. He was placed upon his right side, so as to make the wound dependent, to facilitate drainage, and keep the viscera in contact with the injured parietes, with a view of preventing further hemorrhage and looking to the possible adhesion of the injured parts to the peritoneum. After consultation it was deemed improper to remove the clothing, as such a proceeding would thus increase the dangers. Water was given in small quantities, often repeated. This was necessitated by the extreme thirst from which the patient suffered.

A hypodermic injection of one-eighth of a grain of morphine and one-eightieth grain of atropia was administered to control the pain in the extremities, and as a more permanent stimulant to assist reaction. The place selected for injection was the dorsal aspect of the forearm. This was about 10 A. M., July 2d.

There was but little change in the condition of the patient, either in temperature, respiration, or pulse, until about 11 o'clock when it was determined to repeat the morphine in the dose of one-sixth of a grain, the atropia being omitted. This soon had the effect of modifying the pain and discomfort, and the respiration beeame more frequent and easy. The pulse responded but little to the stimulants. Nausea and vomiting continued at intervals of thirty minutes during the entire day and until 7 P. M., when it became less frequent, with less retching—in fact, being simply a regurgitation of the fluids of the stomach, This condition continued at longer intervals until 6 o'clock the following morning.

At 5.30 P. M., in accordance with a previous understanding with the physicians, the clothing was removed by being cut from the body in such a manner as to prevent any motion or agitation, and to permit the more successful application of dry heat by warm flannels to the entire body, which had been imperfectly accomplished before. Upon examination, a well-defined field of dullness over the region of the wound, thought to be due to hemorrhage in the substance of the liver, along the supposed track of the ball, extended seven and one-half inches antero-posteriorly and five and one-half inches laterally.

The urine was retained until 6 o'clock P. M., when a flexible, velvet-eyed catheter was introduced, and about six ounces of normal urine drawn. During the remainder of his illness the urine was voided without restraint, and frequent careful examinations were made, proving the absence of albumen or other significant abnormal ingredients. A spontaneous evacuation of the bowels took place on July 3d, which was natural in character and free from blood or other foreign matter. After this, and during the entire period of his illness, the President was not subject to diarrhœa, and his movements were either spontaneous or regulated by enemata. The only exception to this was that during the last few days of his illness, occasional small involuntary evacuations took place, which seemed to depend upon the existence of large hemorrhoids, which, from their size and locality, produced dilatation and partial paralysis of the sphincter muscle, the evacuation always occurring in an effort to expel flatus.

At 10 P. M. the pulse was 158, temperature 96.5°, respiration 35, which was the most critical period attending the collapse. At 11:20 P. M. the evidences of reaction began to manifest themselves.

When the pulse had diminished to 120, the temperature had risen to 98° F., and the respiration was 18. The carbolized absorbent cotton which had previously sealed the wound having become displaced, was reapplied.

Until 2 P. M. of July 3d, the variations of pulse were comparatively slight, ranging from 104 to 120, the respiration being normal.

The patient slept at short intervals, generally arousing with an effort at regurgitation of the contents of the stomach, but otherwise expressed a feeling of comfort and gave evidences of rest. During the night he seemed to be refreshed and was comparatively free from pain. There was no time after my first visit, up to this period, that the patient was not perfectly rational, and often made brief, pertinent inquiries as to the character of the wound and his condition.

At the evening consultation, July 2d (7 P. M.), the opinion was expressed by some of the medical gentlemen invited to the case, that internal hemorrhage had taken place, and that he would not survive the night, and expressed these views to the council. The symptoms of profound collapse were so grave that Surgeon-General Wales was induced to express the opinion that the President was dying.

The consultations heretofore referred to were, as a matter of course, held in the adjoining room. Only three or four physicians of the number present were invited to visit the bedside on each occasion to make personal examinations, to verify the reported progress, and enable them to intelligently advise the council.

The gentlemen invited by me to visit the bedside were Surgeon-General Wales, Surgeon J. J. Woodward, and Dr. Reyburn. On that occasion the opinion was expressed that the field of dulness heretofore referred to, the boundaries of which were well defined, was thought to be due to hemorrhage in the substance of the liver, from the passage of the ball into or through it. The opinion obtained, and was so expressed to the council, that internal hemorrhage was then taking place, and that the extreme prostration and feebleness of the respiration were due to that cause, and that the President would not survive the night.

There was some oozing of dark venous blood during the entire night, sufficient to saturate the carbolized cotton and stain the bed. On the following morning the hemorrhage had entirely ceased, and the dressings became adherent to the skin.

All the physicians visited the White House at 8 A. M., July 3d, for the morning consultation, agreeably to a previous understanding that such should be the case if the President survived the night.

At this consultation, Surgeon-General Barnes and Surgeon Woodward, U. S. A., Dr. Reyburn and Dr. N. S. Lincoln, visited the bedside of the patient with me, with a view of making the necessary examinations, dressing the wound, and of reporting results to the other members of the council. The patient was found with a pulse of 115; the temperature was nearly normal, as was the respiration. He was cheerful, gave evidence of being rested, and made definite inquiries regarding his condition and prospects. The use of morphine hypodermically, in doses of sufficient quantities to control the pain in the extremities, was advised, and it was agreed that the patient should continue to occupy the position on his right side as before directed, so far as was possible ; and that the wound should be exposed only when the dressings became disarranged ; and that their character should not be changed.

Immediately after the consultation, the subject of medical attendance was considered by the President. The only persons present were, besides the President, Mrs. Garfield and myself. He then formally placed himself under my professional care, and requested me to select my counsel, the result of which is well known. He also desired me to individually thank the large number of physicians who had composed the council up to that time, which I accordingly did.

The primary reaction reached the highest point of temperature pulse, and respiration at 2 P. M., on Sunday, July 3d. Slight tympanites was detected, but no pain on pressure, nor any marked rigidity of the abdominal walls. These were the only symptoms which pointed to the existence of peritonitis throughout the whole course of the case, and the spontaneous movement of the bowels, already noted, was an additional evidence that the peritoneum was not involved.

At 10:45 P. M., the pulse had gradually increased in frequency until it reached 120. The temperature remained 100°, and respiration at 20. At this time Dr. D. Hayes Agnew, of Philadelphia, and Dr. Frank H. Hamilton, of New York, were summoned to visit the patient in consultation. Dr. Agnew arrived about 4 o'clock the following morning, July 4th, and Dr. Hamilton at 6 A. M. They were presented to the President formally at the consultation, 8:15 A. M., July 4th, at which time the pulse was 104, temperature 99.4°, and respiration 19. He had passed a comparatively comfortable night, awakening every twenty or thirty minutes, taking water or liquid nourishment in small quantities each time, and dropping quickly to rest. The nausea had quite subsided, and the pain and soreness of the lower extremities was measurably controlled by the administration of morphia, which was continued in quarter-grain doses each evening, administered hypodermically.

A careful review of the case from the time I first saw the President was given to these gentlemen, with the request that they, with the data before them, examine the case thoroughly, as though it was their own, and freely express their views of the character and gravity of the injury and the course of treatment of the case up to that time. I also gave them a detailed account of the explorations made in the wound, and the unsettled convictions as then held as to the course of the missile and the organs involved in the injury. They individually examined the wound with great care. These examinations consisted in the introduction, in different directions, of probes, flexible bougies, in order, if possible, to determine the course of the ball. With the evidences developed by this personal examination, together with the complete history of the shooting of the President, and the progress of the symptoms for the first forty-seven hours, they proceeded to discuss the probable course of the ball and organs involved, viz., whether it passed directly forward into or through the liver, or was deflected backward at a right angle so as to involve the spinal column, or downward behind the peritoneum toward the pelvic cavity. Carefully weighing all the evidences, the more prominent symptoms upon which the diagnosis was based are presented in the following order: The relative position of the assassin to the President at the time of the shooting, the direction of the ball through the tissues, so far as safe exploration could determine, gradual subsidence or modification of pain and hyperæsthesia of the feet and scrotum; the repeated unsucessful efforts to pass a probe or flexible instrument more than one-half-inch in any direction beyond the fractured rib, except in a direction downward, a little forward and anterior to the twelfth rib, a distance of about two inches. The fact also was considered that explorations had twice been made with the finger—one by myself soon after I reached the injured President, and subsequently by Surgeon-General Wales of the navy, on the occasion of the consultation on the evening of July 2d; and in each instance it was found impossible to successfully explore by that means beyond the inner border of the fractured rib, so as to determine with accuracy the course of the ball, or even the condition of the tissues indicated, by the end of the finger. Nor did they underestimate the significance of the profound shock, nor the unusual period of collapse which followed and seemed to point to extensive lesion of important viscera. However, that the kidneys, intestine, and peritoneum were not immediately involved, was made patent by the unrestrained passage of normal urine at proper intervals, the spontaneous movement from the bowels of natural fæces, the frequent discharge of flatus, and the absence of other symptoms of peritonitis. With all these facts before them it was impossible to determine positively the course taken by the ball. The indications pointed to a downward course of the ball into the pelvic cavity. Upon careful consideration of the foregoing facts and of the opinions expressed by the distinguished counsel, we were inclined to recede from the opinion at first adopted, regarding the supposed passage of the ball through the liver. The propriety of making extensive incisions and dissections so as to explore the fractured ribs and remove as much as might be necessary to reveal the true course of the ball, was duly considered. But the opinion was maintained that the favorable progress of the President thus far did not warrant any interference, and, further, such an operation would seriously complicate the case and diminish the prospects of recovery. The facts revealed by the autopsy confirm the wisdom of the course pursued. With this view all the surgeons concurred.

The subsequent history of the case, which proved that the liver, kidneys, the intestines, and the large vessels had escaped serious injury, as well as the gradual subsidence of the nervous disturbance of the lower extremities, the almost entire absence of pain in the back or that portion of the body in which the track existed, together with a pus-sac which dissected its course down behind the peritoneum into the right iliac fossa, was but corroborative, and naturally misled our judgments into an erroneous diagnosis.

On the evening of July 4th, the pain, hyperæsthesia, and vomiting had nearly disappeared, soreness of the feet supervening and continuing for some days.

The case progressed, with slight fluctuations, up to July 23d, when a rigor occurred at 7 P.M., followed by a pulse of 124, respiration 26, and temperature 104°. F. Two days previous to this a pus-sac was observed in the common integument, extending down below the twelth rib toward the erector spinæ muscle, and underneath the latissimus dorsi, and was carefully evacuated by gentle pressure into the original opening on the occasion of each dressing. We did not feel satisfied that this superficial and limited collection of pus, which was so readily evacuated, was the principal cause of the aggravation of the symtoms present. However, a free incision was made into the pus-sac, which afforded a more direct and dependent channel to the fractured rib, from which a small fragment of bone was removed.

Pressure made backward and upward upon the abominal wall, between the umbilicus and the anterior spine, gave exit to a flow of peculiarly white and firm pus. I remarked at the time to the council that the appearance of this pus gave assurance that it had never been exposed to the air, and must have come from a deep-seated source.

After this operation the improvement was not as prompt as we had reason to expect, and on the 26th of July the opening between the fractured ends of the eleventh rib was enlarged, and a small detached portion was removed. This facilitated the discharge of pus, and, as a result, a more uniform condition of the symtoms were maintained until about August 6th, when slight febrile exacerbations were observed which continued to be manifest until the operation was made to afford a more free passage of pus from the supposed track of the ball. The necessity of the operation was more plainly developed by passing a flexible catheter through the opening previously made, which readily coursed toward the crest of the ilium, a distance of about seven inches. This cavity was evacuated twice daily, by passing through the catheter, previously inserted in the track, an aqueous solution of permanganate of potash from a small hand-fountain, slightly elevated, the water and pus returning and escaping at the opening externally. The indications for making a point of exit in the dependent portion of this pus-sac were urgent, and on August 8th the operation was performed by extending the incision previously made, downward and forward through the skin, subcutaneous fascia, external and internal oblique muscles, to a sinus or pus-channel. The exposed muscle contained a considerable number of minute spiculæ of bone. Upon carrying a long, curved director through the opening between the fractured rib downward to the point of incision, there was a deeper channel which had not been exposed by the operation thus far, and the incision was carried through the transversalis muscle and transversalis fascia, opening into the deeper track and exposing the end of the director. A catheter was then passed into the portion of the track below the incision, a distance of three and one-half inches, and in a direction near the anterior superior spinous process of the ilium. The President was etherized during this operation.

A comparatively uniform condition of temperature, pulse and respiration continued until August 14th, when nausea, vomiting and general prostration occurred, with an increase of pulse to 108, temperature 100.8° F., and respiration 19—the pulse continuing to increase, although the temperature remained nearly normal up to August 17th, when food was again retained by the stomach. When the previous attack of vomiting took place, August 14th, the stomach was placed at physiological rest, and resort was had to rectal alimentation until August 17th, when the function of the stomach was gradually reestablished and the enemata discontinued.

On August 18th a slight tumefaction of the right parotid gland was noticeable, unaccompanied by pain or tenderness on pressure, until the suppurative period was established, when mental disturbance, vomiting, restlessness and jactitation supervened ; nor was there any increase of temperature, local or systemic, to indicate the probability of its metastatic origin. The parotitis presented many of the characteristics of an ordinary carbuncle, and was unaccompanied by any other abscesses in the adjoining tissue. During the progress of the parotitis facial paralysis occurred, and continued, with slight improvements until the time of his death. When the climax of suppuration was reached, a free discharge of laudable pus followed, with a rapid abatement of the more urgent symptoms, and after the separation of the slough (which was limited in extent) reparation was rapid and complete throughout the entire suppurating surface, as well as in the several incisions which had been previously made to liberate the pus. These lesions had entirely healed at the time of death, except an opening behind and below the right ear, referred to in the autopsy.

It was a marked feature during this whole period of parotid suppuration, that there was no associate systemic disturbance. The question of malarial complication was discussed at this time, but it must be remembered that quinine had been given in tonic doses much of the time ; and occasionally when periodicity was noticeable, sedative doses were administered for a a period of twenty-four hours at a time.

On August 19th a small slough was discharged from the lower pus-track, when the flexible catheter was readily passed downward a distances of twelve inches toward the right iliac fossa. This channel was kept free from accumulations by passing into it carbolic or permanganate water from the hand-fountain heretofore described, at the same time carefully withdrawing the catheter, so as to avoid undue distention of the track.

During the latter part of August a number of pustules of suppurating acne appeared in the axillæ, and later, four or five on the surface of the body. They were superficial, numbering five or six in each axilla, and about the size of large peas ; they were opened as soon as suppuration took place, healed without recurrence, and are believed to have been due to the septic condition of the system. The small carbuncle mentioned in the report of the autopsy was doubtless referrable to the same cause. The above were the only suppurating surfaces, excepting the incisions made into the wound, and four small superficial bedsores formed on the sacrum, which were observed during the President's illness.

The subject of the removal of the President to a more salubrious locality had been discussed for several days, and was urgently presented at the consultation on August 25th. The majority of the council, with myself, considered that his removal at this time would be attended with very great hazard. The hope, however, was expressed that the President might be sustained until suppuration was established in the parotid, and the constitutional disturbances incident thereto had subsided, when it would be possible to remove him. Stimulents were given in doses ʒ vj. with ʒ ij. of beef-tea, occasionally introducing ℥ j. of beef peptones, alternated with the yolk of an egg. These measures undoubtedly contributed largely to his sustention during this period of continued gastric disturbance.

Our efforts were rewarded on August 26th by a free discharge of pus from the external auditory canal; also in the mouth. It was believed that the pus which discharged in the mouth dissected its way along the course of Steno's duct. There being rigidity of the masseter muscle, the jaw was fixed so as to preclude the possibility of opening the mouth sufficiently for a satisfactory examination. A tenacious mucus was secreted from that side in large quantities, and occasioned great annoyance. The patient during this period was occasionally wandering in his mind, especially after rousing from sleep. When his attention was fixed by an attendant, his mental condition seemed to be comparatively perfect.

An interesting fact connected with the inflammation of the mucous membrane of the mouth was that it extended by continuity to the pharynx, larynx, trachea and bronchi. The physical signs developed the fact that acute bronchial catarrh was the sequel. Hypostatic congestion of the lungs was observed for some weeks before, more extensive on the right than the left, because of decubitus. On the right side it extended to the sixth rib posteriorly, while on the left side it was comparatively slight. An improved condition was maintained with a free-suppurating condition of the parotid and marked reduction of the tumefaction of the gland.

Finally, it was decided by the majority of the surgeons that the President should be removed to the sea-shore. The details as to the precautions taken to secure a safe transit were minute in every particular, and every provision was made to meet any emergency that might arise in the course of the journey—even preparations for his removal from the train to suitable places on the road had been previously selected, in case evidences of exhaustion should become manifest.

His transfer from the Executive Mansion to the cars was made with the least possible disturbance, without accident, and with perfect satisfaction and comfort to the patient. During the journey his pulse and temperature were taken from time to time, and frequent examinations made to determine the effect of the motion at different rates of speed. The minimum of unpleasant motion seemed to be secured at a rate of about sixty miles an hour. During the last hour of his journey he showed symptoms of fatigue, which would have prevented a longer journey, had such been required to reach his destination. His pulse increased, the countenance became slightly anxious, and the temperature measurably exalted at the period to which I allude.

He was transferred from the cars to the Elberon cottage without accident, the pulse at 10 P. M. reaching 124, temperature 101.6°. The morning of September 7th, his pulse had fallen to 106, temperature 98.4°, respiration 18. The President expressed great satisfaction that he had arrived at the sea-shore, and, notwithstanding the heat of the two succeeding days, it made but little impression upon the distinguished patient, the pulse, temperature, and respiration continuing the same until September 15th, when his pulse slightly increased in the evening, so that it occasionally reached 120 during the night.

After his arrival at Elberon there was an extension of the bronchial catarrh into the ramifications of the bronchi of the right lung, and limited broncho-pneumonia followed.

I should mention here a fact well known, that the President was so much pleased with his improvement that he expressed that the number of his professional attendants should be reduced. Accordingly

Drs. Barnes, Woodward, and Reyburn retired from the case, leaving Elberon the morning of September 8th.

September 17th, at 11 A. M., a severe rigor occurred of half an hour's duration, followed by a sharp rise in temperature. At 12 M. the pulse was 120, temperature 102° F., and respiration 24. The mental disturbances were more noticeable during the febrile rise, but the stomach was able to retain the nourishment and stimulants, which were given at regular intervals in the form of milk-punch. The chill was accompanied by severe pain over the anterior mediastinum, and the President said to me that it was similar to what he understood as angina pectoris. It is evident that this pain, which occurred on several occasions at intervals of six to twelve hours prior to his death, was occasioned by first a rupture of the aneurismal sac, and the progressive dissection, at irregular intervals, of the blood into the surrounding tissue, until finally it burst into the peritoneum.

A febrile rise was very marked by twelve noon of the 17th, attended wtth great anxiety of· countenance, the temperature falling to 98° F., the lowest point of normal range, the pulse being, however, steadily at 102, and rather feeble. While there was, in my judgment, an absence of typical metastatic abscesses to produce this symptom, there was a profound expression of gravity in his condition that was not commensurate with the systemic disturbance, and which prevented my absence, even for a few moments at a time. I remarked to Dr. Agnew: "I am in constant fear of some danger impending. We may have a terrible outburst, possibly in the shape of a cardiac thrombus." I said to·members of the family: "There is a gravity in this case that portends serious trouble."

At 6 P. M. of the 18th there was another chill, accompanied with pain as before. The febrile rise continued until midnight, the pulse varying from 112 to 130.

At 8 A. M., September 19th, the pulse was 106 and feeble; temperature, 108.8°, and all the conditions unfavorable. In half an hour afterward there was still another chill, followed by febrile rise and sweating, and also with pain as before. During the periods of chill and fever he was more or less unconscious. He passed all day in comparative comfort, and at 8.30 in the evening his pulse was 108, respiration 20, and temperature evidently a little lower than normal.

At 10.10 P. M. I was summoned hastily to the bed-side, and found the President in an unconscious and dying condition, pulseless at the wrist, with extreme pallor, the eyes opened and turned upward, and respiration eight per minute, and gasping. Placing my finger upon the carotid, I could not recognize pulsation ; applying my ear over the heart, I detected an indistinct flutter, which continued until 10.35, when he expired. The brave and heroic sufferer, the nation's patient, for whom all had labored so cheerfully and unceasingly, had passed away.

Soon after the President expired, it became necessary to make arrangements for an autopsy, so as to present to the profession, in a definite manner, the track of the ball and the parts involved ; also to ascertain the immediate cause of death. I deemed it proper to invite Surgeon-General Barnes, and Surgeon J. J. Woodward, U. S. A., and Dr. Robert Reyburn, of Washington, D. C., who were formerly associated in the case, to take part in the autopsy, and also invited, at the instance of Dr. Woodward, Dr. Lamb, of the Army Medical Museum, for the same purpose. The former gentlemen arrived at Elberon, N. J., about 3.45 P. M., when the post-mortem examination was commenced. Dr. A. H. Smith, of New Jersey and New York, and temporarily at Elberon, was also invited.

The most important points revealed by the autopsy, which are to be considered by the profession, are :

*First.*—Would the condition of the President, immediately after his injury, have justified a more thorough exploration of the wound, or would such a procedure have been safe at any time before primary reaction was established ?

*Second.*—Was his transfer to the executive mansion timely and properly made ?

*Third.*—Were the best and most judicious means instituted to secure prompt reaction ?

*Fourth.*—After reaction was comparatively complete on the 3d of July, and when there occurred spontaneous evacuations of normal urine and alvine evacuations, and an absence of any evidence of internal hemorrhage or peritonitis, would further exploration have been necessary, especially when it is considered that the probable reopening of the lacerated vessels would induce hemorrhage ?

*Fifth.*—Were the surgeons then in attendance justified in deferring any further exploration until the arrival of the distinguished counsel on the morning of July 4th ?

*Sixth.*—At the consultation, July 4th, and after it was proved to be impossible to follow the track of the ball any considerable distance beyond the fractured rib, would an operation have been justifiable necessitating an incision through the soft parts and a removal of a portion of the rib, so as to develop the track.

*Seventh.*—In the light of modern military surgery, which teaches the readiness with which leaden balls become encysted, would an operation at any time for removal of the missile have been justified unless there was some evidence of the missile being a source of irritation?

*Eighth.*—Considering carefully the condition of the President during the entire period of his illness, and the facts revealed by the autopsy, would not any operation for the purposes before mentioned have placed the President's life in great jeopardy, and, at best, have hastened the time of his death, without affording any signal relief?

*Ninth.*—Was the treatment of the case as presented proper, and did it or not prolong his life to the utmost limit?

*Tenth.*—Was the mistaken diagnosis a natural result of the conditions present, and to have developed a correct diagnosis would not operative procedures have ensued?

*Eleventh.*—If we had known the exact course and locality of the ball, and the organs injured in its passage, should the treatment have been modified in any particular?

The artistic drawings which accompany this history, will, I trust, facilitate its study, and their accuracy is not only attested by myself, but by Prof. Faneuil D. Weisse, M. D., and Dr. Geo. F. Shrady, of New York, both of whom visited Washington, on my invitation, to study the case, and make thorough and personal examination of the specimens preserved, with a view of verifying the facts in its history.

These drawings were made by Mr. Max Cohn, of New York, who came to Washington, D. C., for that purpose, at my request.

They very satisfactorily illustrate the point of impact and course of the ball, and the pathological conditions which followed, and upon which the diagnosis and treatment were based.

I desire to say, in a brief review of the leading facts as to the general conduct of the case, that it has been apparent to the medica reader thas my prognosis was favorable, and notwithstanding the mutations I augured a successful termination. It is but justice to myself to state that my prognosis was based on a lesion of minor importance. Had our diagnosis been correct, modern surgery should have conducted the case to a successful termination. I believe the medical profession, whom I address, will bear me out that the prognosis was correct if the diagnosis had been also correct. I was not always able, during the progress of the case, to account for many of the more profound symptoms, and yet could not succeed in learning of any more extensive or complicated lesions than were first suspected. I desire to make the inquiry whether more extensive explorations could have been safely made, or whether the condition presented—a knowledge of the relative position of the patient to the assassin, the character of the missile, and the condition of the lesion and symptoms which follow—would have directed the investigation toward the actual track and lodgment of the ball, the track of the ball presenting a course of entrance downward and forward to the point of impingement upon the eleventh rib, and being then deflected to the left at almost a right angle, passing behind the kidney, perforating the intervertebral cartilage and first lumber vertebræ anterior and to the left of the kidney, and finding its lodgement below the left extremity of the pancreas, wounding in its track the splenic artery. I would ask if any known instrument or means of exploration has ever been presented to the profession capable of tracing before the death of said patient the course of this bullet? Also whether the conditions could have been improved or mitigated, or his life preserved longer by any other line of treatement; whether, in view of the facts, modern conservative surgery could offer anything more for the comfort or recovery of the illustrious patient.

It is proper to state, in conclusion, that most approved antiseptic dressings were used during the entire progress of the case.

The following is the report of the autopsy, to which reference has been made:

*Record of the Post-mortem Examination of the Body of President J. A. Garfield, made September* 20, 1881, *commencing at* 4:30 *P. M., eighteen hours after death, at Francklyn Cottage, Elberon, New Jersey.*

Present and assisting: Dr. D. W. Bliss; Surgeon General J. K. Barnes, U. S. Army ; Surgeon J. J. Woodward, U. S. Army ; Dr. Robert Reyburn, Dr. Frank H. Hamilton, Dr. D. Hayes Agnew, Dr. Andrew H. Smith, of Elberon (and New York), and Acting Assistant Surgeon D. S. Lamb, of the Army Medical Museum, Washington, D. C.

Before commencing the examination, a consultation was held by these physicians, in a room adjoining that in which the body lay, and it was unanimously agreed that the dissection should be made by Dr. Lamb, and that Surgeon Woodward should record the observations made. It was further unanimously agreed that the cranium should not be opened. Surgeon Woodward then proposed that the examination should be conducted as follows :

That the body should be viewed externally, and any morbid appearances existing recorded ; that a catheter should then be passed into the wound, as was done during life, to wash it out, for the purpose of assisting to find the position of the bullet; that a long incision should next be made from the superior extremity of the sternum to the pubes, and this crossed by a transverse one just below the umbilicus ; that the abdominal flaps thus made should then be turned back and the abdominal viscera examined ; that after the abdominal cavity was opened the position of the bullet should be ascertained, if possible, before making any further incision ; and that, finally, the thoracic viscera should be examined.

This order of procedure was unanimously agreed to.

The examination was then proceeded with, and the following *external appearances* were observed :

The body was considerably emaciated, but the face was much less wasted than the limbs. A preservative fluid had been injected by the embalmer, a few hours before, into the left femoral artery. The pipes used for the purpose were still in position. The anterior surface of the body presented no abnormal appearances, and there was no ecchymosis or other discoloration of any part of the front of the abdomen.

Just below the right ear, and a little behind it, there was an ova-ulcerated opening, about half an inch in long diameter, from which some sanious pus was escaping, but no tumefaction could be observed in the parotid region.

A considerable number of purpura-like spots were scattered thickly over the left scapula, and thence forward as far as the axilla. They ranged from one-eighth to one-fourth of an inch in diameter, were slightly elevated and furfuraceous on the surface, and many of them were confluent in groups of two to four or more. A similar, but much less abundant, eruption was observed sparsely scattered over the corresponding region on the right side.

An oval excavated ulcer about an inch long, the result of a small carbuncle, was seated over the spinous process of the tenth dorsal vertebra. Over the sacrum there were four small bed-sores, the largest about half an inch in diameter. A few acne pustules, and a number of irregular spots of post-mortem hypostatic congestion were scattered over the shoulders, back, and buttocks. The inferior part of the scrotum was much discolored by hypostatic congestion. A group of hemorrhoidal tumors, rather larger than a walnut, protruded from the anus.

The depressed cicatrix of the wound made by the pistol-bullet was recognized over the tenth intercostal space, three and one-half inches to the right of the vertebral spines. A deep linear incision (made in part by the operation of July 24th, and extended by that of August 8th) occupied a position closely corresponding to the upper border of the right twelfth rib. It commenced posteriorly about two inches from the vertebral spines, and extended forward a little more than three inches. At the anterior extremity of this incision there was a deep, nearly square abraded surface about an inch across.

A well-oiled flexible catheter, fourteen inches long, was then passed into this wound, as had been done to wash it out during life. More resistance was at first encountered than had usually been the case, but after several trials the catheter entered, without any violence, to its full length. It was then left in position, and the body disposed supinely for the examination of the viscera.

The *cranium* was not opened.

A long incision was made from the superior extremity of the sternum to the pubis, followed by a transverse incision crossing the abdomen just below the umbilicus. The four flaps thus formed were turned back and the abdominal viscera exposed. The subcutaneous adipose tissue divided by the incision was little more than one-eighth of an inch thick over the thorax, but was thicker over the abdomen, being about one-fourth of an inch thick along the linea alba, and as much as one-half inch thick toward the outer extremity of the transverse incision.

On *inspection of the abdominal viscera in situ*, the transverse colon was observed to lie a little above the line of the umbilicus. It was firmly adherent to the anterior edge of the liver. The greater omentum covered the intestines pretty thoroughly from the transverse colon almost to the pubes. It was still quite fat, and was very much blackened by venous congestion. On both sides its lateral margins were adherent to the abdominal parietes opposite the eleventh and twelfth ribs. On the left side the adhesions were numerous, firm, well organized, and probably old.* On the right side there were a few similar adhesions, and a number of more delicate and probably recent ones.

A mass of black, coagulated blood covered and concealed the spleen and the left margin of the greater omentum. On raising the omentum it was found that this blood-mass extended through the left lumbar and iliac regions and dipped down into the pelvis, in which there was some clotted blood and rather more than a pint of bloody fluid.† The blood-coagula having been turned out and collected, measured very nearly a pint. It was now evident that secondary hemorrhage had been the immediate cause of death, but the point from which the blood had escaped was not at once apparent.

The omentum was not adherent to the intestines, which were moderately distended with gas. No intestinal adhesions were found other than those between the transverse colon and the liver, already mentioned.

The abdominal cavity being now washed out as thoroughly as possible, a fruitless attempt was made to obtain some indication of the position of the bullet before making any further incision. By pushing

---

* These adhesions, and the firm ones on the right side, as well as those of the spleen, possibly date back to an attack of chronic dysentery, from which the patient is said to have suffered during the civil war.

† A large part of this fluid had probably transuded from the injecting material of the embalmer.

the intestines aside, the extremity of the catheter, which had been passed into the wound, could be felt between the peritoneum and the right iliac fascia; but it had evidently doubled upon itself, and, although a prolonged search was made, nothing could bé seen or felt to indicate the presence of the bullet,either inthat region or elsewhere.

The abdominal viscera were then carefully removed from the body, placed in suitable vessels, and examined *seriatim*, with the following results:

The adhesions between the liver and the transverse colon proved to bound an *abscess-cavity* between the under-surface of the liver, the transverse colon, and the transverse mesocolon, which involved the gallbladder, and extended to about the same distance on each side of it, measuring six inches transversely and four inches from before backward. This cavity was lined by a thick pyogenic membrane, which completely replaced the capsule of that part of the undersurface of the liver occupied by the abscess. It contained about two ounces of greenish yellow fluid—a mixture of pus and biliary matter. This abscess did not involve any portion of the substance of the liver except the surface with which it was in contact, and no communication could be detected between it and any part of the wound.

Some recent peritoneal adhesions existed between the upper surface of the right lobe of the liver and the diaphragm. The *liver* was larger than normal, weighing eighty-four ounces; its substance was firm, but of a pale-yellowish color on its surface and throughout the interior of the organ, from fatty degeneration. No evidence that it had been penetrated by the bullet could be found, nor were there any abscesses or infarctions in any part of its tissue.

The *spleen* was connected to the diaphragm by firm, probably old, peritoneal adhesions. There were several rather deep congenital fissures in its margins, giving it a lobulated appearance. It was abnormally large, weighing eighteen ounces; of a very dark lake-red color both on the surface and on section. Its parenchyma was soft and flabby, but contained no abscesses or infarctions.

There were some recent peritoneal adhesions between the posterior wall of the *stomach* and the posterior abdominal parietes. With this exception no abnormities were discovered in t[illegible] stomach or *intestines*, nor were any other evidences of general or l[illegible]al peritonitis found besides those already specified.

The *right kidney* weighed six ounces, the *left kidney* seven. Just beneath the capsule of the left kidney, at about the middle of its convex border, there was a little abscess one-third of an inch in diameter, and there were three small serous cysts on the convex border of the right kidney, just beneath the capsule ; in other respects the tissue of both kidneys was normal in appearance and texture.

The *urinary bladder* was empty.

Behind the right kidney, after the removal of that organ from the body, the dilated *track of the bullet* was dissected into. It was found that from the point at which it had fractured the right eleventh rib (three and one-half inches to the right of the vertebral spines) the missile had gone to the left, obliquely forward, passing through the body of the first lumbar vertebra and lodging in the adipose connective tissue immediately below the lower border of the pancreas, about two and one-half inches to the left of the spinal column, and behind the peritoneum. It had become completely encysted.

The track of the bullet between the point at which it had fractured the eleventh rib and that at which it entered the first lumbar vertebra was considerably dilated, and the pus had burrowed downward through the adipose tissue behind the right kidney, and thence had found its way between the peritoneum and the right iliac fascia, making a descending channel which extended almost to the groin. The adipose tissue behind the kidney in the vicinity of this descending channel was much thickened and condensed by inflammation. In the channel, whieh was found almost free from pus, lay the flexible catheter introduced into the wound at the commencement of the autopsy; its extremity was found doubled upon itself, immediately beneath the peritoneum, reposing upon the iliac fascia, where the channel was dilated into a pouch of considerable size. This long descending channel, now clearly seen to have been caused by the burrowing of pus from the wound, was supposed during life to have been the track of the bullet.

The last dorsal, together with the first and second lumbar vertebra and the twelfth rib, were then removed from the body for more thorough examination.

When this examination was made, it was found that the bullet had penetrated the first lumbar vertebra in the upper part of the right side of its body. The aperture by which it entered involved the intervertebral cartilage next above, and was situated just below and anterior to the intervertebral foramen, from which its upper margin was about one-fourth of an inch distant. Passing obliquely to the left, and forward to the upper part of the body of the first lumbar vertebra, the bullet emerged by an aperture, the centre of which was about one-half inch to the left of the median line, and which also involved the intervertebral cartilage next above. The cancellated tissue of the body of the first lumbar vertebra was very much comminuted and the fragments somewhat displaced. Several deep fissures extended from the track of the bullet into the lower part of the body of the twelfth dorsal vertebra. Others extended through the first lumbar vertebra into the intervertebral cartilage between it and the second lumbar vertebra. Both this cartilage and that next above were partly destroyed by ulceration. A number of minute fragments from the fractured lumbar vertebra had been driven into the adjacent soft parts.

It was further found that the right twelfth rib also was fractured at a point one and one-fourth inch to the right of the transverse process of the twelfth dorsal vertebra; this injury had not been recognized during life.

On sawing through the vertebra, a little to the right of the median line, it was found that the spinal canal was not involved by the track of the ball. The spinal cord, and other contents of this portion of the spinal canal, presented no abnormal appearances. The rest of the spinal cord was not examined.

Beyond the first lumbar vertebra, the bullet continued to go to the left, passing behind the pancreas to the point where it was found. Here it was enveloped in a firm cyst of connective tissue, which contained, besides the ball, a minute quantity of inspissated, somewhat cheesy pus, which formed a thin layer over a portion of the surface of the lead. There was also a black shred adherent to a part of the cyst-wall, which proved, on microscopical examination, to be the remains of a blood-clot. For about an inch from this cyst the track of the ball behind the pancreas was completely obliterated by the healing process. Thence, as far backward as the body of the first lumbar vertebra, the track was filled with coagulated blood, which extended on the left into an irregular space rent in the adjoining adipose tissue behind the peritoneum and above the pancreas. The blood had worked its way to the left, bursting finally through the peritoneum behind the spleen into the abdominal cavity. The rending of the tissues by the extravasation of this blood was undoubtedly the cause of the paroxysms of pain which occurred a short time before death.

This mass of coagulated blood was of irregular form, and nearly as large as a man's fist. It could be distinctly seen from in front through the peritoneum, after its sight behind the greater curvature of the stomach had been exposed by the dissection of the greater omentum from the stomach, and especially after some delicate adhesions between the stomach and the part of the peritoneum covering the blood-mass had been broken down by the fingers. From the relations of the mass as thus seen, it was believed that the hemorrhage had proceeded from one of the mesenteric arteries, but as it was clear that a minute dissection would be required to determine the particular branch involved, it was agreed that the infiltrated tissues and the adjoining soft parts should be preserved for subsequent study.

On the examination and dissection made in accordance with this agreement, it was found that the fatal hemorrhage proceeded from a rent, nearly four-tenths of an inch long, in the main trunk of the splenic artery, two and one-half inches to the left of the cœliac axis. This rent must have occurred at least several days before death, since the everted edges in the slit in the vessel were united by firm adhesions to the surrounding connecting tissue, thus forming an almost continuous wall bounding the adjoining portion of the blood-clot. Moreover, the peripheral portion of the clot in this vicinity was disposed in pretty firm concentric layers. It was further found that the cyst below the lower margin of the pancreas, in which the bullet was found, was situated three and one-half inches to the left of the cœliac axis.

Besides the mass of coagulated blood just described, another, about the size of a walnut, was found in the greater omentum, near the splenic extremity of the stomach. The communication, if any, between this and the larger hemorrhagic mass could not be made out.

The examination of the *thoracic viscera* resulted as follows:

The *heart* weighed eleven ounces. All the cavities were entirely empty except the right ventricle, in which a few shreds of soft, reddish, coagulated blood adhered to the internal surface. On the surface of the mitral valve there were several spots of fatty degeneration; with this exception the cardiac valves were normal. The muscular tissue of the heart was soft, and tore easily. A few spots of fatty degeneration existed in the lining membrane of the aorta just above the semilunar valves, and a slender clot of fibrin was found in the aorta, where it was divided, about two inches from these valves, for the removal of the heart.

On the right side slight pleuritic adhesions existed between the convex surface of the lower lobe of the lung and the costal pleura, and firm adhesions between the anterior edge of the lower lobe, the pericardium, and the diaphragm. The *right lung* weighed thirty-two ounces. The posterior part of the fissure between its upper and lower lobes, was congenitally incomplete. The lower lobe of the right lung was hypostatically congested, and considerable portions, especially toward its base, were the seat of broncho pneumonia. The bronchial tubes contained a considerable quantitx of stringy muco-pus; their mucous surface was reddened by catarrhal bronchitis. The lung-tissue was œdematous,* but contained no abscesses or infarctions.

On the left side the lower lobe of the lung was bound behind to the costal pleura, above to the upper lobe, and below to the diaphragm, by pretty firm pleuritic adhesions. The *left lung* weighed twenty-seven ounces. The condition of the bronchial tubes and of the lung-tissue was very nearly the same as on the right side, the chief difference being that the area of the broncho pneumonia in the lower lobe was much less extensive in the left lung than in the right. In the lateral part of the lower lobe of the left lung, and about an inch from its pleural surface, there was a group of four minute are as of gray hepatization, each about one-eighth of an inch in diameter. There were no infarctions and no abscesses in any part of the lung tissue.

The surgeons assisting at the autopsy were unanimously of the opinion that, on reviewing the history of the case in connection with the autopsy, it is quite evident that the different suppurating surfaces and especially the fractured spongy tissue of the vertebra, furnish a sufficient explanation of the septic conditions which existed during life.

---

*A part, at least, of this condition was doubtless due to the extravasation of the injecting fluid used by the embalmer.

About an hour after the post-mortem examination was completed, the physicians named at the commencement of this report assembled for further consultation in an adjoining cottage ; a brief outline of the results of the post-mortem examination was drawn up, signed by all the physicians, and handed to private secretary J. Stanley Brown, who was requested to furnish copies to the newspaper press.

(Signed) D. W. BLISS,
J. K. BARNES,
J. J. WOODWARD,
ROBERT REYBURN,
D. S. LAMB.

As the above report contains paragraphs detailing the observations made at Washington on the pathological specimens preserved for that purpose, the names of Drs. F. H. Hamilton, D. Hayes Agnew, and A. H. Smith, are not appended to it. It has, however, been submitted to them, and they have given their assent to the other portions of the report.—*Medical Record.*

---

### SURGICO-ANATOMICAL STUDY OF THE GUNSHOT WOUND OF PRESIDENT GARFIELD

BY FANEUIL D. WEISSE, M. D.,
Professor of Practical and Surgical Anatomy, Medical Department of the University of the City of New York.

After the autopsy on the remains of the late President, performed September 20, 1881, the following seemed to have been the course of the fatal ball after it entered the President's body, and the same was verified by actual dissections :

*The ball entered opposite the tenth intercostal space, about four inches to the right of the median line of the back. It ranged in a direction forward and downward, inclining a little from right to left. It perforated to the plane of the ribs through the skin, subcutaneous tissue, fascia, the latissimus dorsi, seratus posticus inferior, and sacro-lumbalus muscles. It impinged upon the eleventh rib (the most movable of all the ribs), which it crowded to a plane anterior to that of the twelfth. It produced*

*a comminuted fracture of the eleventh rib. The impact of the ball on the eleventh rib caused it to turn on its axis, and from there it was deflected to the left. It perforated the eleventh external intercostal muscle and the sub-pleural portion of the diaphragm just above the right ligamentum arcuatum externum. It tracked through the connective and adipose tissue between the superior portion of the right kidney and the twelfth rib to the spinal column. It pierced the attachment of the right psoas magnus muscle to the first lumbar vertebra. It entered the body of the first lumbar vertebra from right to left. It emerged from the left of the spine, pierced the left psoas magnus muscle attachment, and entered a plane of connective and adipose tissue between the left kidney posteriorly and the left half of the pancreas anteriorly. It crossed the posterior surface of the pancreas obliquely to the left and from above downward to its point of lodgement. It wounded the splenic artery in its transit across the pancreas (the splenic artery presenting in the track of the ball, it seemed more than probable that it, and not the mesenteric, would prove, upon a careful dissection, to have been the injured vessel), from which source the final hemorrhage occurred which burst into the peritoneal cavity.*

I visited Washington with Dr. Geo. F. Shrady, upon the invitation of Dr. D.W.Bliss. At that time Dr. Shrady was informed of the above theory and dissections, but Dr. Bliss knew nothing of these anatomical investigations. Dr. Bliss related to Dr. Shrady and myself a detailed history of the President's case from July 2d to the time of his death. The pathological specimens taken from the body were placed at our disposal by Surgeon J. J. Woodward, U. S. A., in charge of the Army Medical Museum (where the specimens are),and we studied them carefully. Drs. Woodward and Lamb had made careful dissections of the pathological specimens, by which were revealed the following conditions, which lack of time had rendered impossible to have been known at the time of the issue of the Elberon autopsy bulletin:

*First.*—The existence of a united fracture of the right twelfth rib.

*Second.*—The entrance of the ball at the right side of the intervertebral fibro-cartilage, between the twelfth dorsal and the first lumbar vertebræ, involving the adjoining portion of the body of the first lumbar vertebra, anterior to the right intervertebral foramen, between the pedicles of the twelfth dorsal and the first lumbar vertebræ.

*Third.*—The transit of the ball through the superior half of the body of the first lumbar vertebra, from right to left and obliquely forward and downward, producing a comminuted fracture of the body of the vertebra.

*Fourth.*—That the intervertebral fibro-cartilage between the first and second lumbar vertebræ had been injured, by the comminution of the body of the vertebra contiguous to it.

*Fifth.*—That the left anterior inferior edge of the body of the twelfth dorsal vertebra was broken away.

*Sixth.*—The evidences of a traumatic aneurism in communication with the splenie artery, at about two and one-half inches from its origin from the cœliac axis.

*Seventh.*—The cyst which contained the ball was found at the inferior border of the external left third of the pancreas posterior to the peritoneum, viz., the posterior inferior layer of the lesser omentum, which becomes the anterior superior layer of the transverse meso-colon.

*Eighth.*—The imperviousness of the track of the ball for an inch or more from the cyst.

The specimen of the abdominal viscera did not present anything bearing upon the location of the collection of pus to the right of the vertebral column described in the Elberon autopsy bulletin.

Reviewing the theory previously deduced, with these additional facts, there was found only this to add:

*In that portion of its course after deflection from the eleventh rib to the spine, the ball grazed the anterior surface of the twelfth rib, producing a simple fracture of it.*

*The transit of the ball through the spine had been defined, but remained virtually the same.*

The injuries inflicted by the ball may be epitomized as follows :

*First.*—A compound comminuted fracture of the eleventh rib.

*Second.*—A compound fracture of the twelfth rib.

*Third.*—A compound comminuted fracture of the body of the first lumbar vertebra, complicated with injury to the intervertebral fibro-cartilages above and below that vertebra, and the breaking off of the border of the twelfth dorsal vertebra.

*Fourth.*—A wound of the splenic artery.

The anatomical reasons for the sequelæ of the above injuries are :

*Sequelæ of the first injury.*—The compound comminuted fracture of the eleventh rib developed the superficial abscess opened by the first operation ; the debris of the comminuted rib—thrown by the deflection of the ball downward into the substance of the muscles of the parietes—was the direct cause of irritation, etc.

*Sequelæ of the third injury.*—The concussion to the spinal column had its expression in the symmetrical pains at the peripheral distributions of the right and left sacral plexuses, below the knees, indicating disturbances of their contributive spinal nerves, which constitute the major portion of the cauda quina within the lumbar region of the column.

The pain in the right inguinal region and of the right side of the scrotum would find its explanation in a special impression upon the right first lumbar nerve—the anterior branches of which are the right ilio-hypogastric and ilio-inguinal nerves distributing thereto—which is in close relation with the first lumbar vertebra.

The comminuted fracture of the lumbar vertebra, the injury to the twelfth dorsal, and the injured intervertebral fibro-cartilages determined a consecutive destructive inflammation, the pus from which drained to the right. The pus, however, did not altogether follow the track of the ball posterior to the right kidney; some of it passed anterior to the right kidney, dissecting its way posterior to the peritoneum.

The pus, taking the posterior course, dissected its way down in the post-visceral and extra-peritoneal (sub-serous) areolar tissue, interior to the transversalis fascia of the abdominal parietes, the process ultimately resulting in the sinus, which extended to the iliac fossa.

The pus taking the anterior course did not have an outlet, and a reservoir of it accumulated between the right kidney posteriorly and the peritoneum covering the liver and the colon anteriorly.

The contiguity of this pus to the liver and colon induced a protecting localized adhesion of the opposed peritoneal surfaces, investing the liver on the one hand, and the hepatic flexure and right half of the transverse colon on the other. These conditions were verified at the autopsy. "An abcess-cavity, six inches by four in dimensions, was found in the vicinity of the gall-bladder, between the liver and the transverse colon, which were strongly adherent. It did not in-

Fig. 1

Fig. 2

Fig. 3

Fig. 4

Fig. 5

Fig. 6

Fig. 7

Fig. 8

PRESIDENT GARFIELD'S CASE.—Pathological Specimens and Anatomical Cuts, showing course of ball.

volve the substance of the liver, and no communication was found between it and the wound." It is possible that there was an error as to the location of this pus; in exposing the abcess *in situ* the adhesions between the liver and colon were progressively separated, and the collection of pus—really behind the posterior plane of the peritoneum, in the right lumbar region—as it bulged forward, gave the semblance of lodgment between the liver and colon.

*Sequelæ of the fourth injury.*—The injury to the splenic artery developed a traumatic aneurism, as appeared in the dissected specimen. The location was such that it obstructed the track of the ball to the left side of the spine, in which position it determined two reparative processes: 1st, the obliteration of the track of the ball to the left of the first lumbar vertebra, which prevented the drainage of pus in that direction. 2d, the lodged ball, sealed hermetically from access of air by the closure of its track, became at once encysted.

The surgical anatomy of this memorable case of gunshot wound admits of the following conclusions:

*First.*—It is a matter of great regret that the autopsy was not made from the back instead of the anterior plane of the body. The bullet entered from behind, and from this direction it should have been followed; after which the internal organs could have been examined for evidences of any remote effects of the wound. Dissections demonstrate, conclusively, the advantages, in this particular case, of such a course, as it would have exposed the lesions and the encysted ball *in situ.*

*Second.*—The impossibility at any time to have safely or even successfully probed the wound so as to find the ball.

*Third.*—The ball as lodged was shut in beneath the abdominal parietes and kidney posteriorly, and the abdominal parietes and spleen laterally and to the left. In this location no operation was warranted for its removal, there having been no evidence of its presence there.

*Fourth.*—Had the point of lodgement of the ball been known, the fact of its not producing any local irritation—the formation of an abscess around it—would have been a positive contra-indication against any operation looking to its removal.

*Fifth.*—If an abscess had formed around the ball instead of its becoming encysted, the pus might have burst into the peritoneal cavity or pointed in the left loin below the kidney, and formed a similar post-visceral and extra-peritoneal sinus to the one that existed on the right side.

The rupture into the peritoneal cavity would probably have been provided against by the occurrence of suitably protecting peritoneal adhesions. Had the sinus formed, it would have afforded a clue to a correct diagnosis; and under these conditions, after an incision had been made to allow the escape of pus at the left side, an exploratory operation, with the sinus as a guide to the ball, might have been warranted, even to the determining its positive location, and possibly its extraction. Indeed, the ball itself might have dropped into the left sinus.

*Sixth.—It was anatomically possible for a ball deflected downward from the eleventh rib to take the same course as did the sinus to the iliac fossa, and there was afforded—by the rapidity with which this sinus formed; the readiness with which the drainage tubes passed; the fact that the incision of the second operation tapped the sinus below the twelfth rib; the fact that the wound of entrance of the ball healed so promptly after the incisions below; the existence early in the case of a point of tenderness in the right iliac fossa and the subsequent recognizable induration there, which gradually diminished—sufficient grounds to warrant the diagnosis that was arrived at and maintained up to the time of the death of the patient, especially so in the absence of any evidence that the ball had taken a different course.*—Medical Record

### OPERATION FOR NECROSIS.

(Performed before the New Jersey State Dental Society, July 21, 1881.)

BY DR. GEO. A. MILLS, BROOKLYN.

The patient, a robust young man of about thirty years of age, I had not seen until a few hours previous to the operation. The history of the case is briefly this: About eight years ago,while splitting wood, he was struck in the mouth, knocking out, as he supposed, one of he upper incisors. Inflammation set in, followed by a discharge of fetid pus, mostly through an opening into the left nostril. He fell into the hands of a dentist who diagnosed the adjacent tooth to be the cause of the trouble, and extracted it. The patient obtained no relief, and the discharge of pus continued up to the time of the operation. About three months ago, the case came to the attention of Dr. Charles Hubbard, of Brooklyn, who diagnosed the necrosis, but did not discover the cause of it until he learned of the accident above mentioned, and that the tooth which was supposed to have been knocked out had not been found. Ascertaining by probing that it was still remaining in the jaw, he transferred the case to me for operation. I dissected away the gum-tissue attached to the upper lip, making an incision about one and a-half inches long. Dissecting back, I discovered a necrosed condition of the parts continuous to the buried tooth. With a bur in a surgical engine, I enlarged the orifice in the direction of the tooth, which was lying in an oblique direction, nearly half an inch above the apex of the left cuspid, and between the point of the root of the cuspid and the left portion of the base of the nose. It proved, on further investigation, that the tooth had been driven into the transverse process between the two plates of the superior maxilla. By cutting away the portion of the bone to the left of the foreign body, and enlarging the opening sufficiently for its ultimate removal, after stopping the hemorrhage and drying out the cavity, the cutting edge of the tooth was readily discovered. An attempt was made to remove it with forceps, but it slipped back into the enlarged cavity which had been created in the vicinity by diseased action. Two hooked instruments were then employed and its extraction effected. It was quite extensively fractured on the cutting edge, and necrosed some three-eighhts of an inch from the apex back upon the body of the root. One curious circumstance was discovered after its removal, which was, that the gum-tissue surrounding the neck of the tooth when in its normal position, had been partly stripped from its attachment to the process, and carried with the tooth into the location in which it was found, some slight connection with the myxomatous tissue being maintained, which had sustained its normal condition of vitality through all these years. After removing the tooth, the cavity was syringed out with lukewarm salt water, and quite an amount of broken-down tissue was forced out through the opening into the nostril. Dressing with a weak solution of arnica and water, the flaps of the wound were laid together and covered with a pack of cotton. Of the final recovery and entire healthfulness of the parts there is no doubt, and without disfigurement that will not be entirely remedied by the insertion of two artificial teeth. The operation was performed without the administration of any anesthetic, and occupied about three-quarters of an hour.—*Dental Cosmos.*

### GALVANISM IN THE TREATMENT OF HEMORRHOIDS; AND ITS INCIDENTAL EFFECT UPON HABITUAL CONSTIPATION.

We have treated hemorrhoids according to the directions given in standard works, and by injecting carbolic acid into the pile-tumors with the hypodermic syringe. Each of these plans of treatment gave more or less success, when properly carried out. But recently we have given more particular attention to the treatment of hemerrhoids by application of galvanism; and have found this plan of treatment more successful, and far less objectionable, than any other mode we have ever tried. To be brief as possible, we will concisely state the general rules governing us in the treatment of hemorrhoids with galvanism. We use one sponge-faced electrode, applied externally, to the region where most indicated—to the hepatic region over the abdomen, if constipation detains, or to the dorsal region if backache is present, and so on. The rectal electrode is polished metal, nickel-plated, about three or four inches long, three-fourths of an inch in diameter, with wooden handle. If the case is recent, or the parts ulcerated or very sensitive, we apply the positive pole to the hemorrhoids. But if the parts are not tender, and especially if the tumors are old and tough, we apply to them the negative pole. Any late standard works on electro-therapeutics will explain the philosophy of this mode of procedure. In cases of long standing, when the parts are sensitive, we begin the treatment by applying the positive pole, but so soon as the tenderness is relieved, we apply the negative pole, and thus hasten the cure, as the tumors are thereby more rapidly melted down and absorbed.

Preparatory to the application of galvanism to hemorrhoids we always direct the rectum to be well cleansed with warm tar soap suds. The rectal electrode should be anointed with camphorated vaseline. While treating cases of hemorrhoids with galvanism, we have often observed that the attendant constipation was relieved, incidentally, and the relief thus brought about was more permanent than when carthartics were used. Many cases of hemorrhoids occur in females who suffer from some ailment in the generative organs. In the treatment of such cases of hemorrhoids, we have often observed that local treatment of the generative organs was more rapidly successful when simultaneous with the use of galvanism. We never use a current unpleasantly strong to the patient, or any but the constant current. Length of seances fifteen to thirty minutes, two or three times a week. Duration of treatment, to effect a thorough cure, two weeks to two months. In all cases the following ointment is a valuable adjuvant to the galvanic treatment, and more especially if extreme tenderness or ulceration obtains :

℞ Iodoform, in fine-powder.
Ergotin.
Pow. nutgalls.
Pow. camphor............................āā ℨ ss
Pow. opii.
White wax.
Vaseline................................āā ℨ i
Cocoanut butter.............................℥ i.

A convenient and effective mode of applying the ointment to the piles, is through a glass tube, similar to a glass tube uterine applicator, fitted with a piston. An application of this ointment just after each electrical seance, and every night just before retiring to bed, will expedite the cure and add to the comfort of the patient. We administer such drugs as may be indicated in each case, but mainly rely upon galvanism to cure the hemorrhoids ; and usually little else is required. We deem it unnecessary to enlarge upon this important subject, though the theme is tempting, and fruitful to the practical electro-therapeutist. The ideas that naturally cluster about the hints we have given, wonld cover all we have to say of importance.—*Q. C. Smith, M D., in Nashville Journal of Medicine and Surgery.*

### IS THERE A SPECIFIC URETHRITIS?

In a "special article" in the September number of the *New York Medical Journal and Obstetrical Review* Dr. P. Albert Morrow handles the question of the specific or non-specific nature of gonorrhœa. After a fair statement and a close analysis of the arguments for and against specificity, he concludes that the position of the *virulists* rests altogether upon pure hypothesis, and is wholly untenable, while all the facts—experimental, clinical, and pathological—are overwhelmingly in favor of the non-specific character of gonorrhœal inflammation. When we apply the gauge of specificity to gonorrhœa it corresponds to none of the conditions of an undoubtedly specific inflammation. No artificial production of any disease belonging to this group is possible ; a specific disease is the product alone of a specific poison. Gonorrhœa, on the contrary, may be due to a variety of causes—contagious, irritant (mechanical or chemical), diathetic, etc. Again, in all specific diseases there is between the time of infection and the first expression of the disease a period of incubation. No incubation, properly so called, characterizes gonorrhœa. A drop of this same gonorrhœal pus, which may require two or three days to excite suppuration of the urethra, will develop such effect in a few hours when applied to the conjunctiva, showing that the so-called incubation depends not upon the quality of the exciting cause, but upon the susceptibility of the mucous membrane. Another distinctive peculiarity of this group is that a single attack of the disease confers almost complete security from another attack—a peculiarity precisely the opposite of what is observed of gonorrhœa. The morbid poison of a specific inflammation, once in action, continues until the textural predisposition to its special stimulus is exhausted. The patient is incapable of regenerating the poison or of being affected by it when exposed anew. Both of these conditions are negatived in the clinical history of gonorrhœa. Finally, specific inflammation determines special pathological changes and demands special treatment. Identical pathological processes are met with in urethritis from various causes, and the most radical of virulists treat all urethral inflammations alike.

## Figures and Tables

**Figure f1:**
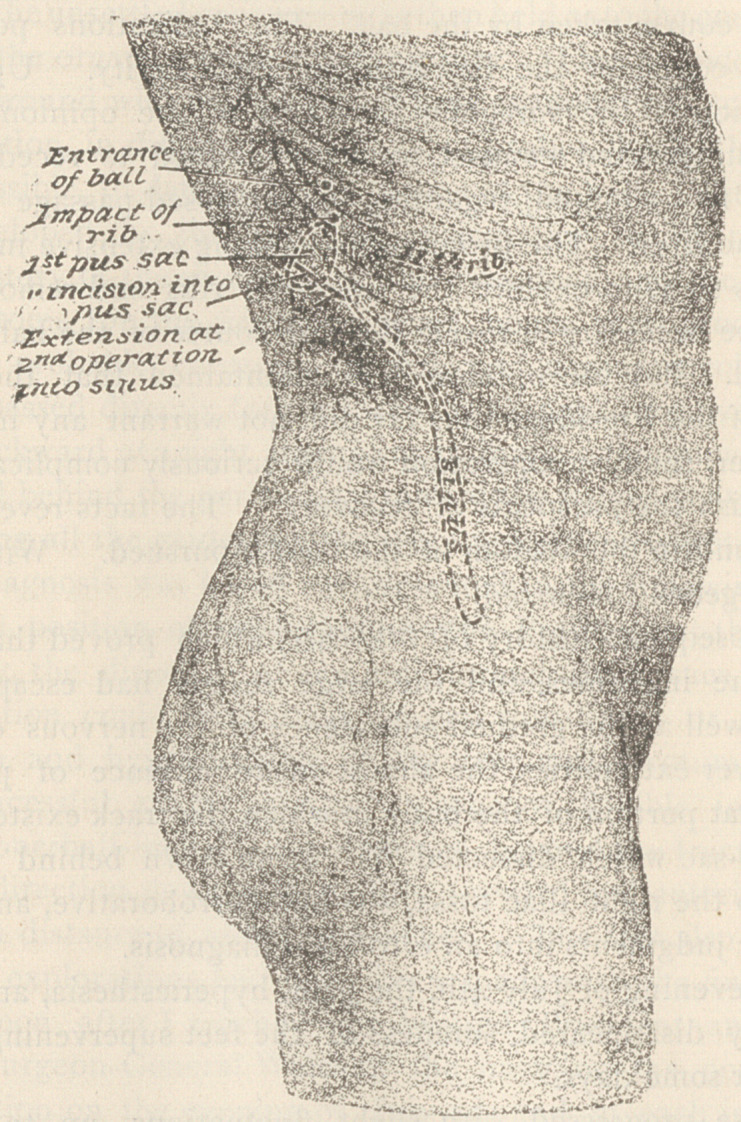


**Fig.1 Fig.2 Fig.3 Fig.4 Fig.5 Fig.6 Fig.7 Fig.8 f2:**